# *Akkermansia muciniphila* alleviates abdominal aortic aneurysms via restoring CITED2 activated by EPAS1

**DOI:** 10.1128/iai.00172-24

**Published:** 2024-08-29

**Authors:** Siqing Wang, Hang Shi, Yue Cheng, Lei Jiang, Yang Lou, Manish Kumar, Mingfei Sun, Xianze Shao, Xuan Zhao, Baichun Wang

**Affiliations:** 1 Department of Cardiovascular Surgery, The Fourth Affiliated Hospital of Harbin Medical University, Harbin, Heilongjiang, China; University of Pennsylvania, Philadelphia, Pennsylvania, USA

**Keywords:** abdominal aortic aneurysms, *Akkermansia muciniphila*, EPAS1, CITED2, vascular smooth muscle cells

## Abstract

Abdominal aortic aneurysm (AAA) is a life-threatening cardiovascular disease that has been linked to gut microbiome dysbiosis. Therefore, this study aims to investigate the effects of *Akkermansia muciniphila* (*Am*) on AAA mice and the biomolecules involved. AAA mice were generated using angiotensin II (Ang II), and 16sRNA sequencing was used to identify an altered abundance of microbiota in the feces of AAA mice. Vascular smooth muscle cell (VSMC) markers and apoptosis, and macrophage infiltration in mouse aortic tissues were examined. The abundance of *Am* was reduced in AAA mouse feces, and endothelial PAS domain-containing protein 1 (EPAS1) was downregulated in AAA mice and VSMC induced with Ang II. *Am* delayed AAA progression in mice, which was blunted by knockdown of EPAS1. EPAS1 was bound to the Cbp/p300-interacting transactivator 2 (CITED2) promoter and promoted CITED2 transcription. CITED2 reduced VSMC apoptosis and delayed AAA progression. Moreover, EPAS1 inhibited macrophage inflammatory response by promoting CITED2 transcription. In conclusion, gut microbiome dysbiosis in AAA induces EPAS1-mediated dysregulation of CITED2 to promote macrophage inflammatory response and VSMC apoptosis.

## INTRODUCTION

Abdominal aortic aneurysm (AAA), a localized dilatation of the infrarenal aorta, results from alterations in the aortic wall structure, including thinning of the media and adventitia because of the loss of vascular smooth muscle cells (VSMCs) and degradation of the extracellular matrix (1). Currently, the only treatment option for AAA is open or endovascular surgical repair, and there is an unmet clinical need to develop medical therapies for small AAA that prevent the progressive expansion and rupture of the aneurysm (2).

The gut microbiota has been recently revealed to play a critical role in AAA formation, and regulation of the microbiota or the immune system might represent a therapeutic approach for AAA (3). For instance, the levels of *Oscillospira*, *Coprococcus*, *Faecalibacterium prausnitzii*, *Alistipes massiliensis*, and *Ruminococcus gnavus* were increased in the angiotensin II (Ang II)-induced AAA mice, whereas those of *Akkermansia muciniphila* (*Am*), *Allobaculum*, and *Barnesiella intestinihominis* were increased in mice with saline (4). In the present study, *Am* was also identified to be the most abundant microbiota in AAA mice induced with Ang II by using 16s rRNA sequencing (5). Consistently, *Am* has been reported to inhibit the formation of AAA and repair tissue damage in mice (6), indicating the possible therapeutic effect. However, the molecular mechanism involved has not been well characterized.

By combining multiple database queries, we obtained endothelial PAS domain-containing protein 1 (EPAS1) as a candidate target of *Am* in AAA. EPAS1, known as hypoxia-inducible factor-2 alpha (HIF-2A), serves as a transcription factor participating in many cellular pathways and has been revealed to regulate the function of SMC in pulmonary hypertension (7). More importantly, EPAS1 has been identified as one of the crucial transcription factors that influence AAA formation in the circulatory system of aged people (8). Wit regard to its connection to microbiota, the gut microbiota has been revealed to produce metabolites that suppress EPAS1, a master transcription factor of intestinal iron absorption, thus contributing to decreased intestinal iron absorption by the host (9). However, whether EPAS1 expression controlled by *Am* is involved in the treatment of AAA has not been previously examined. Moreover, HIF-1 has long been reported to activate the expression of Cbp/p300-interacting transactivator 2 (CITED2) in VSMC (10). CITED2 has been reported to limit the development of atherosclerotic plaques by inhibiting the inflammatory response of macrophages (11). In this study, we provide evidence that CITED2 is a putative target of EPAS1 and dissect that EPAS1, induced by *Am*, limits the development of AAA *in vivo* by activating the expression of CITED2.

## RESULTS

### Reduced abundance of *Am* is identified in the feces of mice with AAA

**Fig 1:**
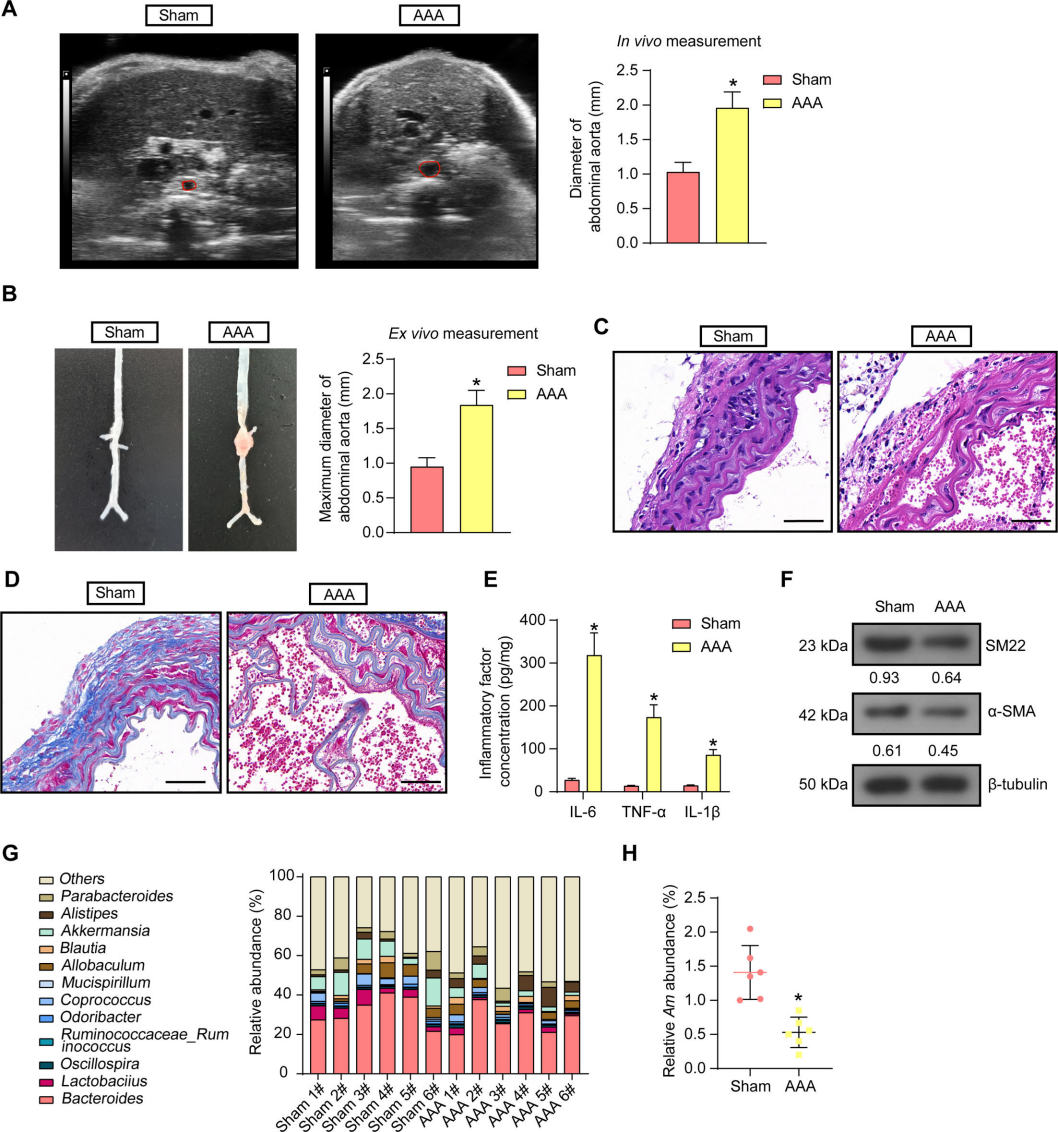
The abundance of *Am* is decreased in AAA mouse feces. (**A**) *In vivo* measurement of abdominal aortic diameter in mice by Doppler ultrasonography. (**B**) *Ex vivo* measurement of the maximum diameter of abdominal aorta in mice. (**C**) The aortic pathology in AAA mice was observed using HE staining. Scale bar, 50 µm. (**D**) The collagen deposition in the aorta of AAA mice was observed using Masson staining. Scale bar, 50 µm. (**E**) IL-6, TNF-α, and IL-1β levels in aortic tissue homogenates were measured using ELISA. (**F**) The protein expression of SM22 and α-SMA, VSMC contractile markers, in the aorta of AAA mice was assessed using Western blot analysis. (**G**) Differences in gut microbiota at the genus level in the feces of sham-operated mice and AAA mice were analyzed using 16s rRNA sequencing. (**H**) Differences in *Am* abundance at the species level in the feces of sham-operated mice and AAA mice were analyzed using 16s rRNA sequencing. *n* = 6/group; *P < 0.05. Data were presented as mean ± SD and analyzed using unpaired *t*-tests or two-way ANOVA.

To investigate the involvement of gut microbiome dysbiosis on the progression of AAA, we first established AAA mice with Ang II for 4 weeks, and Doppler ultrasonography demonstrated that Ang II induction resulted in significant dilatation of the suprarenal aorta in mice (Fig. 1A). The abdominal aorta was isolated from dissected mice. The diameter of the abdominal aorta was measured *ex vivo*. It was found that the abdominal aorta in the renal region was significantly protruded after Ang II treatment (Fig. 1B). HE staining of mouse aorta showed that connective tissues, endothelial cells, and collagen fibers were neatly aligned in the sham-operated mice, whereas the aortic wall of AAA mice was severely damaged, with degradation of elastin fibers and proliferation of collagen fibers (Fig. 1C). Disruption of the aortic collagen network and reduction of collagen deposition in AAA mice were observed using Masson staining (Fig. 1D). Enzyme-linked immunosorbent assay (ELISA) of interleukin (IL)-6, tumor necrosis factor (TNF)-α, and IL-1β levels in aortic tissue homogenates revealed a significant increase in the contents of these inflammatory factors as a result of Ang II induction (Fig. 1E). Western blot detection of VSMC contractile markers showed that Ang II treatment reduced the expression of SM22 and α-SMA (Fig. 1F). Differences in the abundance of gut microbiota in fecal samples from AAA mice and sham-operated mice were analyzed using 16s rRNA sequencing, and the results showed a significantly altered gut microbiota of AAA mice compared with sham-operated mice (Fig. 1G). Among them, we noted the reduced abundance of *Akkermansia* at the genus level in the gut microbiota of AAA mice. Analysis of *Akkermansia* at the species level revealed that *Am* was significantly reduced in abundance in AAA (Fig. 1H).

### *Am* gavage can alleviate AAA in mice induced with Ang II

**Fig 2:**
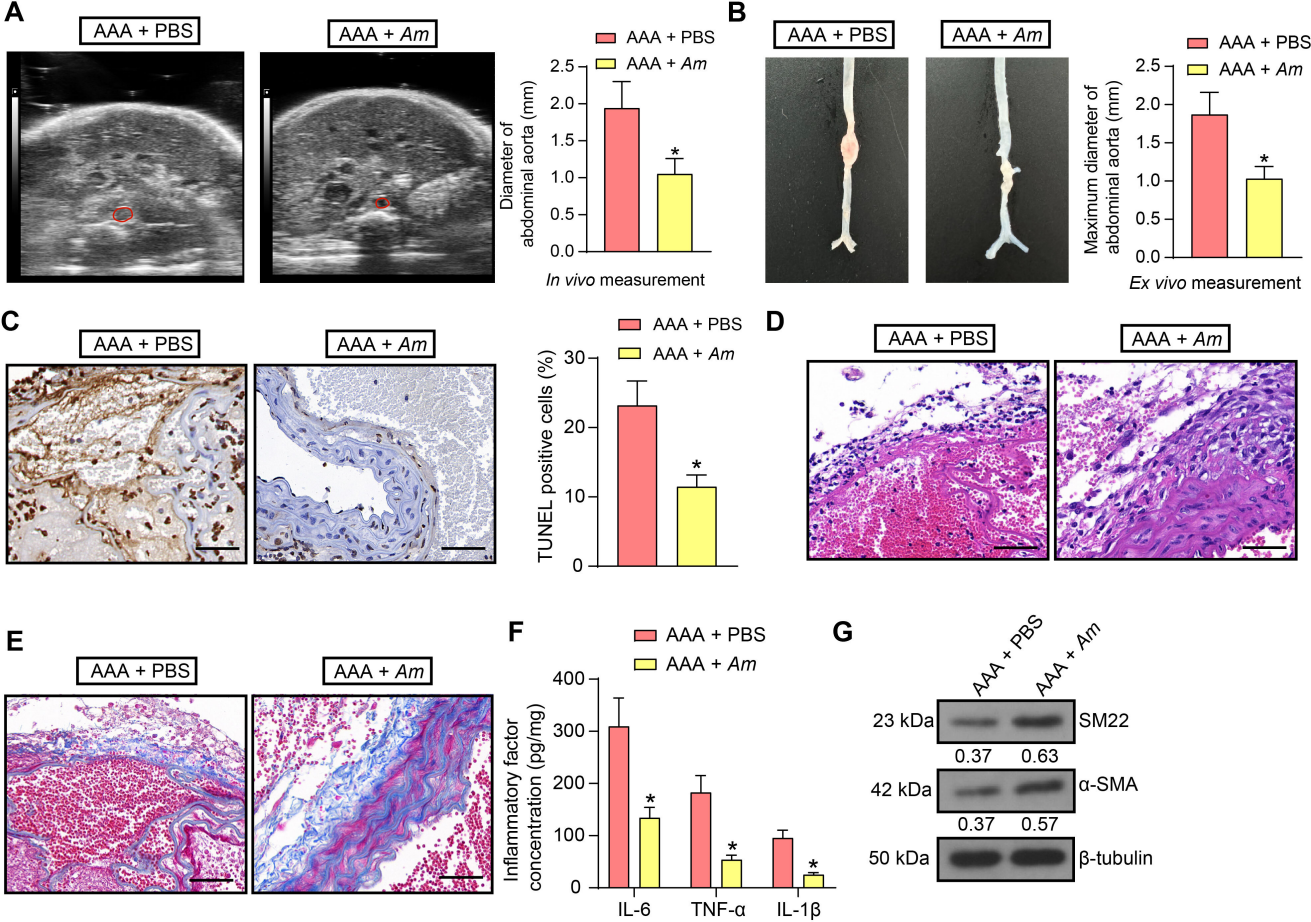
*Am* gavage represses Ang II-induced AAA formation. (**A**) *In vivo* measurement of abdominal aortic diameter in mice treated with *Am* or control PBS by Doppler ultrasonography. (**B**) *Ex vivo* measurement of the maximum diameter of abdominal aorta in mice treated with *Am* or control PBS. (**C**) VSMC apoptosis in AAA mice treated with *Am* or control PBS was analyzed using TUNEL. Scale bar, 50 µm. (**D**) The aortic pathology in AAA mice treated with *Am* or control PBS was observed using HE staining. Scale bar, 50 µm. (**E**) The collagen deposition in the aorta of AAA mice treated with *Am* or control PBS was observed using Masson staining. Scale bar, 50 µm. (**F**) IL-6, TNF-α, and IL-1β Levels in aortic tissue homogenates were measured using ELISA. (**G**) The protein expression of SM22 and α-SMA, VSMC contractile markers, in the aorta of AAA mice treated with *Am* or control PBS, was assessed using Western blot analysis. *n* = 6/group; *P < 0.05. Data were presented as mean ± SD and was analyzed using unpaired *t*-tests or two-way ANOVA.

To demonstrate *in vivo* that *Am* has a therapeutic effect against AAA, we induced AAA mice with Ang II, followed by *Am* gavage. Both *in vivo* ultrasonography (Fig. 2A) and *ex vivo* measurement showed that oral administration of *Am* was able to alleviate the aortic dilatation in mice (Fig. 2B). Terminal deoxynucleotidyl transferase (TdT)-mediated dUTP nick end labeling (TUNEL) assay of VSMC apoptosis revealed that *Am* reduced VSMC apoptosis in the aortic tissues of AAA mice (Fig. 2C). *Am* was observed to alleviate aortic wall damage, elastic fiber degradation, and collagen fiber proliferation by HE staining (Fig. 2D). Masson staining showed that oral administration of *Am* increased collagen deposition in the aorta (Fig. 2E). The results of ELISA and Western blot demonstrated that *Am* partially overcame inflammatory response (Fig. 2F) and augmented the expression of VSMC contractile markers in AAA mice (Fig. 2G).

### *Am* rescues the decrease in EPAS1 expression in aortic tissues of mice induced by Ang II

**Fig 3:**
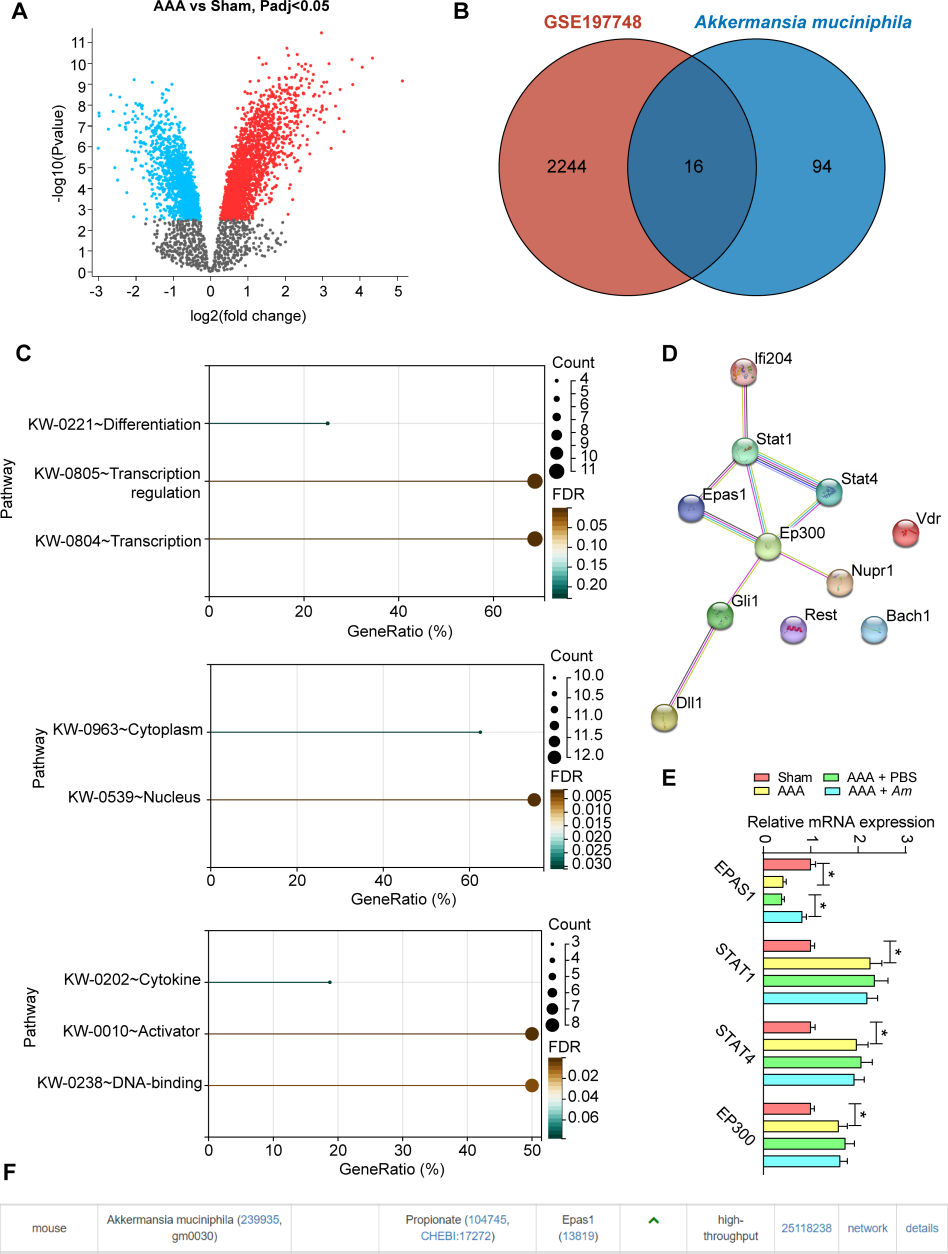
Ang II-induced decrease in EPAS1 expression in AAA mice. (**A**) Differentially expressed genes between AAA and normal mouse aortic tissues in the GSE197748 data set. (**B**) The intersection of differentially expressed genes in data set GSE197748 and prediction results of microbe–target and its metabolite–target from the gutMGene v1.0 data set. (**C**) Functional annotations for intersecting genes at DAVID Bioinformatics Resources. (**D**) PPI network of intersecting genes mainly involved in transcription regulation. (**E**) EPAS1, STAT1, STAT4, and EP300 mRNA expressions in the aortic tissues of AAA mice were assessed using RT-qPCR. (**F**) Metabolite propionate produced by *Am* promotes EPAS1 expression. *n* = 6/group; *P < 0.05. Data were presented as mean ± SD and were analyzed using a two-way ANOVA.

To dissect the possible alteration of molecular mechanisms underlying *Am* in AAA, we downloaded the predicted results of microbe–target and its metabolite–target relationship of *Am* in the gutMGene v1.0 database (https://bio-computing.hrbmu.edu.cn/gutmgene/) and analyzed the differentially expressed genes between AAA mouse aortic tissues and normal mouse aortic tissues in the GSE197748 data set (Fig. 3A). The intersection of the two was taken, which showed a total of 16 intersecting genes (Fig. 3B). We analyzed the functional annotations of the intersecting genes at DAVID Bioinformatics Resources (https://david.ncifcrf.gov/), and the results showed that the intersecting genes are mainly involved in the biological process in transcription regulation, transcription, and differentiation. The intersecting genes are localized in both the nucleus and cytoplasm regarding cellular components and exert molecular functions, including activator, DNA-binding, and cytokines (Fig. 3C). Therefore, we hypothesized that the intersecting genes are mainly related to the transcription regulation during AAA. A protein–protein interaction (PPI) network was generated in the String database using the intersecting genes that are mainly involved in transcription regulation. We noticed a PPI dominated by EPAS1, STAT1, STAT4, and EP300 (Fig. 3D). RT-qPCR was conducted to examine the expression of four transcription factors in the aortic tissues of AAA mice. The expression of EPAS1 was significantly reduced, whereas the expression of STAT1, STAT4, and EP300 was significantly increased in response to Ang II modeling. By contrast, *Am* only rescued EPAS1 expression in the aortic tissues of AAA mice (Fig. 3E). In the gutMGene v1.0 database, we found that propionate, a metabolite produced by *Am*, can promote EPAS1 expression (Fig. 3F). EPAS1 was therefore selected for further analysis.

### EPAS1 knockdown promotes VSMC apoptosis and AAA progression in mice

**Fig 4:**
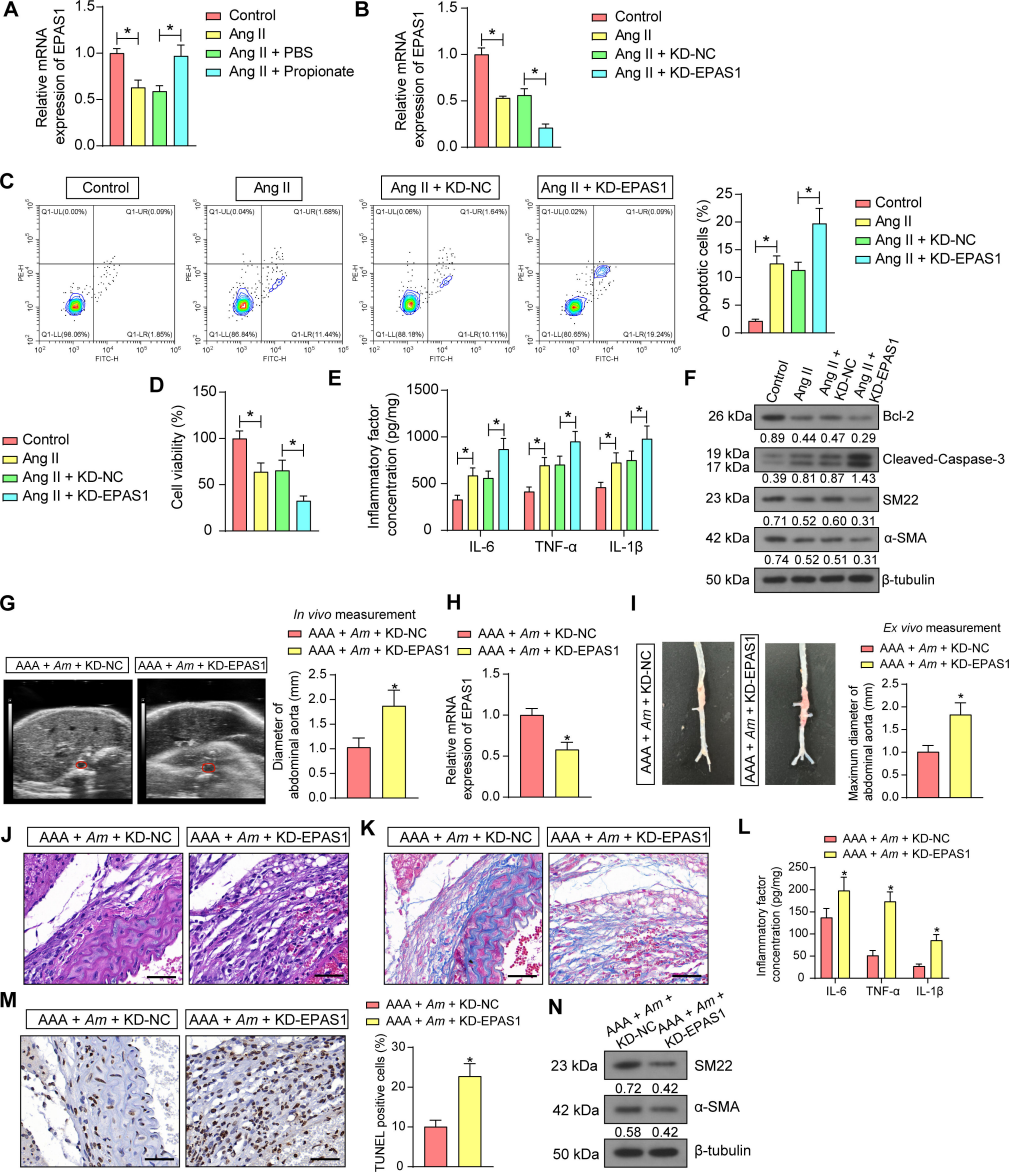
Knockdown of EPAS1 increases VSMC apoptosis and reverses the therapeutic effect of *Am* on AAA in mice. (**A**) Effect of treatment with Ang II and propionate on the expression of EPAS1 mRNA in VSMC assessed using RT-qPCR (*n* = 3 independent experiments). (**B**) EPAS1 mRNA expression in VSMC treated with EPAS1 knockdown and Ang II treatment was assessed using RT-qPCR (*n* = 3 independent experiments). (**C**) Effect of Ang II treatment and knockdown of EPAS1 on apoptosis in VSMC detected by flow cytometry (*n* = 3 independent experiments). (**D**) Effects of Ang II treatment and knockdown of EPAS1 on VSMC viability detected by CCK-8 (*n* = 3 independent experiments). (**E**) The release of inflammatory factors IL-6, TNF-α, and IL-1β in VSMC supernatants was detected by ELISA (*n* = 3 independent experiments). (**F**) Effects of Ang II treatment and knockdown of EPAS1 on the expression of apoptosis-related proteins and VSMC contractile markers in VSMC detected by Western blot (*n* = 3 independent experiments). (**G**) *In vivo* measurement of abdominal aortic diameter in mice treated with KD-NC or KD-EPAS1 by Doppler ultrasonography. (**H**) The mRNA expression of EPAS1 in the aortic tissues of AAA mice was examined using RT-qPCR. (**I**) *Ex vivo* measurement of the maximum diameter of abdominal aorta in mice treated with KD-NC or KD-EPAS1. (**J**) The aortic pathology in AAA mice treated with KD-NC or KD-EPAS1 was observed using HE staining. Scale bar, 50 µm. (**K**) The collagen deposition in the aorta of AAA mice treated with KD-NC or KD-EPAS1 was observed using Masson staining. Scale bar, 50 µm. (**L**) IL-6, TNF-α, and IL-1β levels in aortic tissue homogenates were determined using ELISA. (**M**) VSMC apoptosis in AAA mice treated with KD-NC or KD-EPAS1 was analyzed using TUNEL. Scale bar, 50 µm. (**N**) The protein expression of SM22 and α-SMA, VSMC contractile markers, in the aorta of AAA mice treated with KD-NC or KD-EPAS1, was assessed using Western blot analysis. *n* = 6/group; *P < 0.05. Data were presented as mean ± SD and analyzed using unpaired *t*-tests, one-way, or two-way ANOVA.

We used RT-qPCR to examine the impact of treatment with Ang II and propionate on the expression of EPAS1 mRNA in VSMC and found that the expression of EPAS1 mRNA in VSMC was reduced by treatment with Ang II. After propionate treatment, EPAS1 expression was largely restored (Fig. 4A). Therefore, we constructed VSMC with EPAS1 knockdown (KD-EPAS1, KD-NC as control) and induced them with Ang II. RT-qPCR results showed that KD-EPAS1 further reduced the expression of EPAS1 in the presence of Ang II treatment (Fig. 4B).

By using flow cytometry, we found that Ang II treatment caused VSMC apoptosis, and knockdown of EPAS1 resulted in increased apoptosis in VSMC cells (Fig. 4C). The results of the CCK-8 assay for cell viability showed Ang II-induced reduction in cell viability, and knockdown of EPAS1 further decreased VSMC viability (Fig. 4D). It was demonstrated by ELISA that Ang II produced an inflammatory response in VSMC, and knockdown of EPAS1 increased the contents of IL-6, TNF-α, and IL-1β (Fig. 4E). Finally, apoptosis-related proteins and VSMC contractile markers were detected by Western blot, and it was found that Ang II reduced the expression of the Bcl-2, SM22, and α-SMA and augmented the expression of cleaved-caspase-3, which was exacerbated by knockdown of EPAS1 (Fig. 4F).

To further probe the effects of EPAS1 *in vivo*, lentivirus-encapsulated knockdown of EPAS1 plasmid (named KD-EPAS1 as well, KD-NC as control) was delivered in mice, followed by Ang II induction and *Am* gavage. Before mouse euthanasia, aortic dilatation deteriorated in mice injected with KD-EPAS1 using Doppler ultrasound monitoring (Fig. 4G). After mouse euthanasia, the knockdown of EPAS1 was first found to be effective in the aortic tissues of mice (Fig. 4H). The diameter of the abdominal aorta was measured *ex vivo*, and knockdown of EPAS1 exacerbated aortic dilatation (Fig. 4I). HE staining revealed that knockdown of EPAS1 markedly aggravated the damage to the aortic wall, degradation of elastin fibers, and proliferation of collagen fibers (Fig. 4J). It was observed using Masson staining that knockdown of EPAS1 resulted in reduced collagen deposition and more severe damage to the collagen network (Fig. 4K). Elevated levels of IL-6, TNF-α, and IL-1β in the aortic tissues were also found after knockdown of EPAS1 (Fig. 4L). Finally, enhanced VSMC apoptosis (Fig. 4M) occurred concomitantly with reduced protein expression of SM22 and α-SMA (Fig. 4N).

### EPAS1 promotes transcription of CITED2

**Fig 5:**
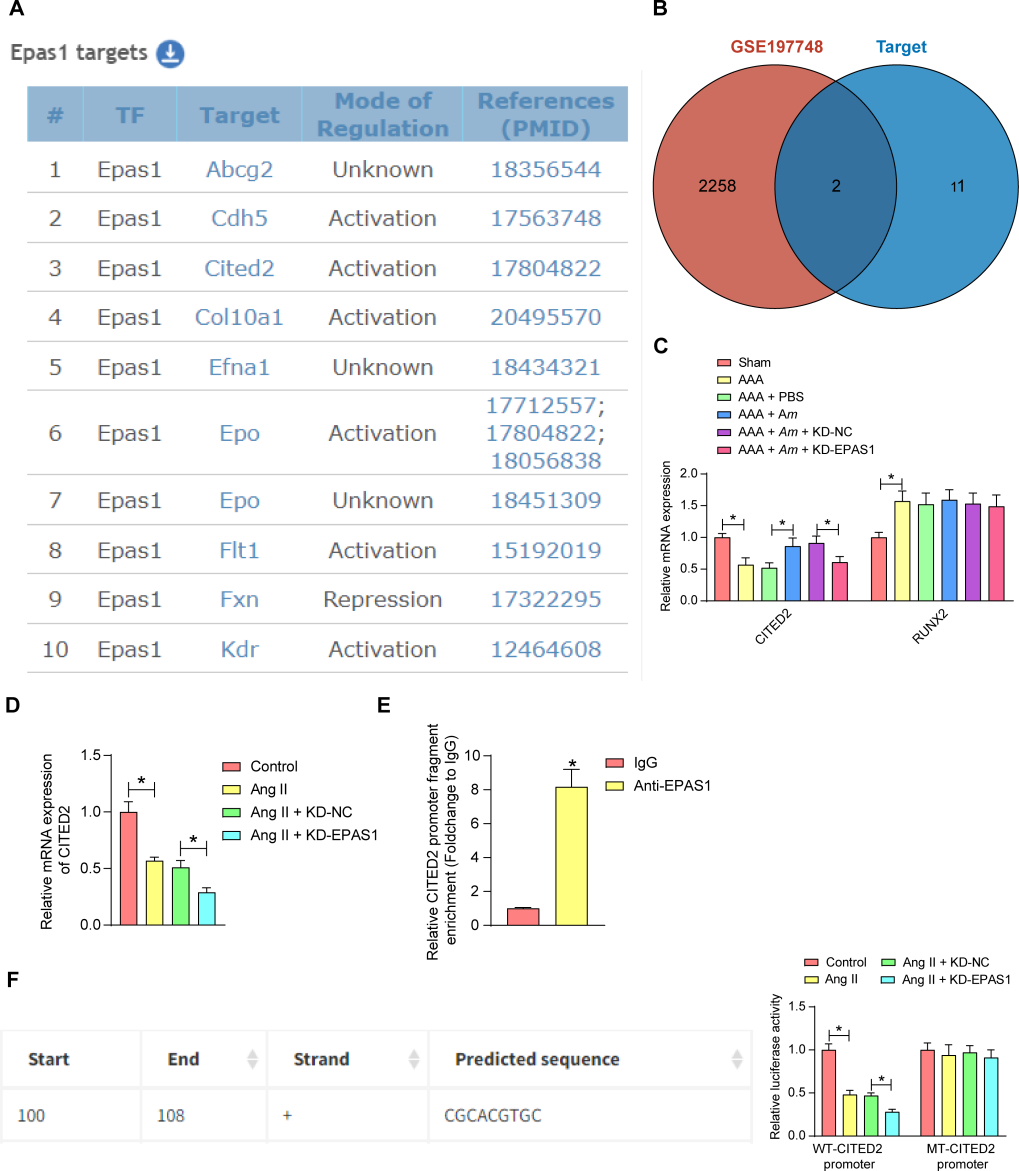
EPAS1 mediates transcriptional activation of CITED2. (**A**) Transcriptional targets predicted to be regulated by EPAS1 in mice in the TRRUST database. (**B**) The intersection of predicted transcriptional targets of EPAS1 and differentially expressed genes in the GSE197748 dataset. (**C**) Effect of Ang II, *Am* treatment, and knockdown of EPAS1 on CITED2 and RUNX2 mRNA expression in mouse abdominal aortic tissues detected by RT-qPCR. (**D**) Effects of Ang II treatment and knockdown of EPAS1 on CITED2 mRNA expression in VSMC detected by RT-qPCR (*n* = 3 independent experiments). (**E**) The binding of EPAS1 and CITED2 promoter was analyzed using ChIP (*n* = 3 independent experiments). (**F**) Transcriptional regulation of CITED2 by EPAS1 was analyzed using a dual-luciferase reporter assay (*n* = 3 independent experiments). *n* = 6/group; *P < 0.05. Data were presented as mean ± SD and analyzed using unpaired *t*-tests one-way, or two-way ANOVA.

To explore the mechanisms by which EPAS1 plays a regulatory role in AAA, the transcriptional targets regulated by EPAS1 in mice were predicted in the TRRUST database (https://www.grnpedia.org/trrust/) (Fig. 5A), which were intersected with the differentially expressed genes in the GSE197748 data set. Only two intersecting genes, CITED2 and RUNX2, were found (Fig. 5B). The expression of CITED2 and RUNX2 mRNA in abdominal aortic tissues of mice treated with Ang II, *Am*, and knockdown of EPAS1 was assessed using RT-qPCR. The downregulation of CITED2 in AAA was restored by *Am* gavage and reduced again by the knockdown of EPAS1, whereas the upregulation of RUNX2 in abdominal aortic tissues of Ang II-induced mice was not altered by *Am* gavage and EPAS1 inhibition (Fig. 5C). Therefore, CITED2 was selected for further analysis.

Consistently, the decline of CITED2 in VSMC induced by Ang II was also further decreased by knockdown of EPAS1 (Fig. 5D). Chromatin immunoprecipitation (ChIP) results showed that EPAS1 was bound to the CITED2 promoter (Fig. 5E). Potential binding sites for EPAS1 on the CITED2 promoter were predicted by JASPAR (https://jaspar.elixir.no/). The dual-luciferase report confirmed that Ang II treatment inhibited the transcriptional activity of the CITED2 promoter containing the binding site with EPAS1, and the knockdown of EPAS1 reduced the transcriptional activity of wild-type (WT) CITED2 promoter. In contrast, Ang II treatment and knockdown of EPAS1 had no significant effect on the transcriptional activity of the mutant (MT)-CITED2 promoter (Fig. 5F).

### EPAS1 inhibits VSMC apoptosis and delays AAA progression in mice by promoting CITED2 transcription

**Fig 6:**
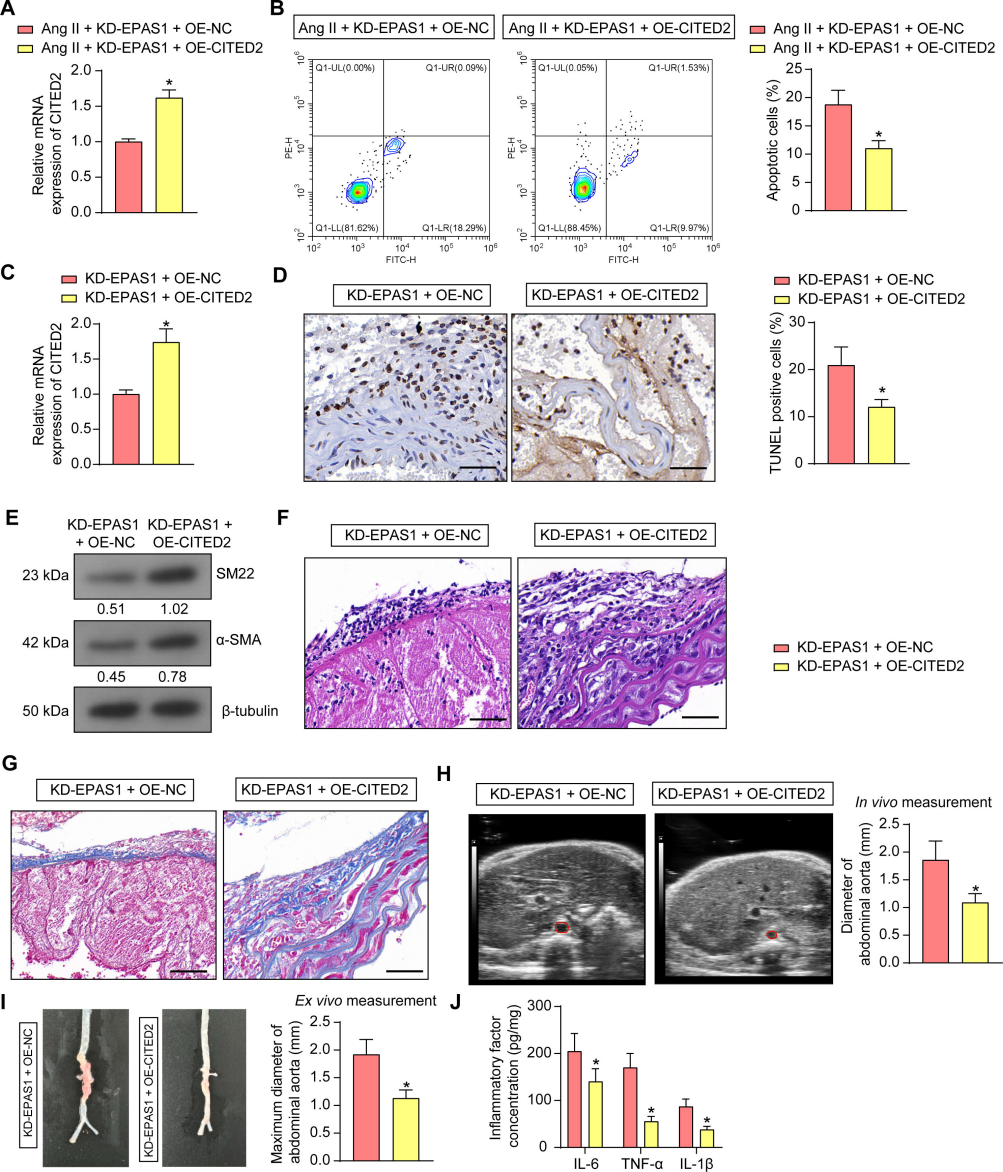
CITED2 overexpression inhibits VSMC apoptosis and delays AAA progression in mice. VSMCs were transfected with KD-EPAS1 and OE-NC or OE-CITED2 (*n* = 3 independent experiments). (**A**) The mRNA expression of CITED2 in VSMC was assessed using RT-qPCR (*n* = 3 independent experiments). (**B**) Effect of overexpression of CITED2 on apoptosis in VSMC detected by flow cytometry. Mice were delivered with lentivirus-encapsulated overexpression CITED2 plasmid based on knockdown of EPAS1, followed by AAA modeling and *Am* gavage. (**C**) The mRNA expression of CITED2 in the aortic tissues of AAA mice was examined using RT-qPCR. (**D**) VSMC apoptosis in AAA mice was analyzed using TUNEL. Scale bar, 50 µm. (**E**) The protein expression of SM22 and α-SMA, VSMC contractile markers, in the aorta of AAA mice was assessed using Western blot analysis. (**F**) The aortic pathology in AAA mice was observed using HE staining. Scale bar, 50 µm. (**G**) The collagen deposition in the aorta of AAA mice was observed using Masson staining. Scale bar, 50 µm. (**H**) *In vivo* measurement of abdominal aortic diameter in mice by Doppler ultrasonography. (**I**) *Ex vivo* measurement of the maximum diameter of the abdominal aorta in mice. (**J**) IL-6, TNF-α, and IL-1β levels in aortic tissue homogenates were determined using ELISA. *n* = 6/group; *P < 0.05. Data were presented as mean ± SD and analyzed using unpaired *t*-tests or two-way ANOVA.

VSMCs were then overexpressed with CITED2 based on the knockdown of EPAS1, followed by Ang II treatment. After verifying the successful overexpression of CITED2 in VSMC (Fig. 6A), we found that the VSMC apoptosis was partially rescued by overexpression of CITED2 (Fig. 6B).

For *in vivo* experiments, the mice were administered with the lentivirus-encapsulated overexpression CITED2 plasmid based on knockdown of EPAS1, followed by AAA modeling and *Am* gavage. RT-qPCR verified that overexpression of CITED2 was effective in the aorta of mice (Fig. 6C). TUNEL assay was used to observe the apoptosis of VSMC, which was decreased after overexpression of CITED2 (Fig. 6D). Western blot detection of VSMC contractile markers revealed that the contractile markers were increased after overexpression of CITED2 (Fig. 6E). Overexpression of CITED2 alleviated aortic wall damage, elastic fiber degradation, and collagen fiber proliferation in AAA mice, as demonstrated by HE staining (Fig. 6F). CITED2 also induced collagen deposition and collagen network recovery in the presence of KD-EPAS1 in AAA mice (Fig. 6G). As for the aortic diameter, both Doppler ultrasound monitoring (Fig. 6H) and *ex vivo* measurement (Fig. 6I) showed that CITED2 upregulation alleviated AAA progression. Finally, inflammatory response in the aorta of mice was attenuated after overexpression of CITED2 (Fig. 6J).

### EPAS1 suppresses macrophage inflammatory responses by promoting CITED2 transcription

**Fig 7:**
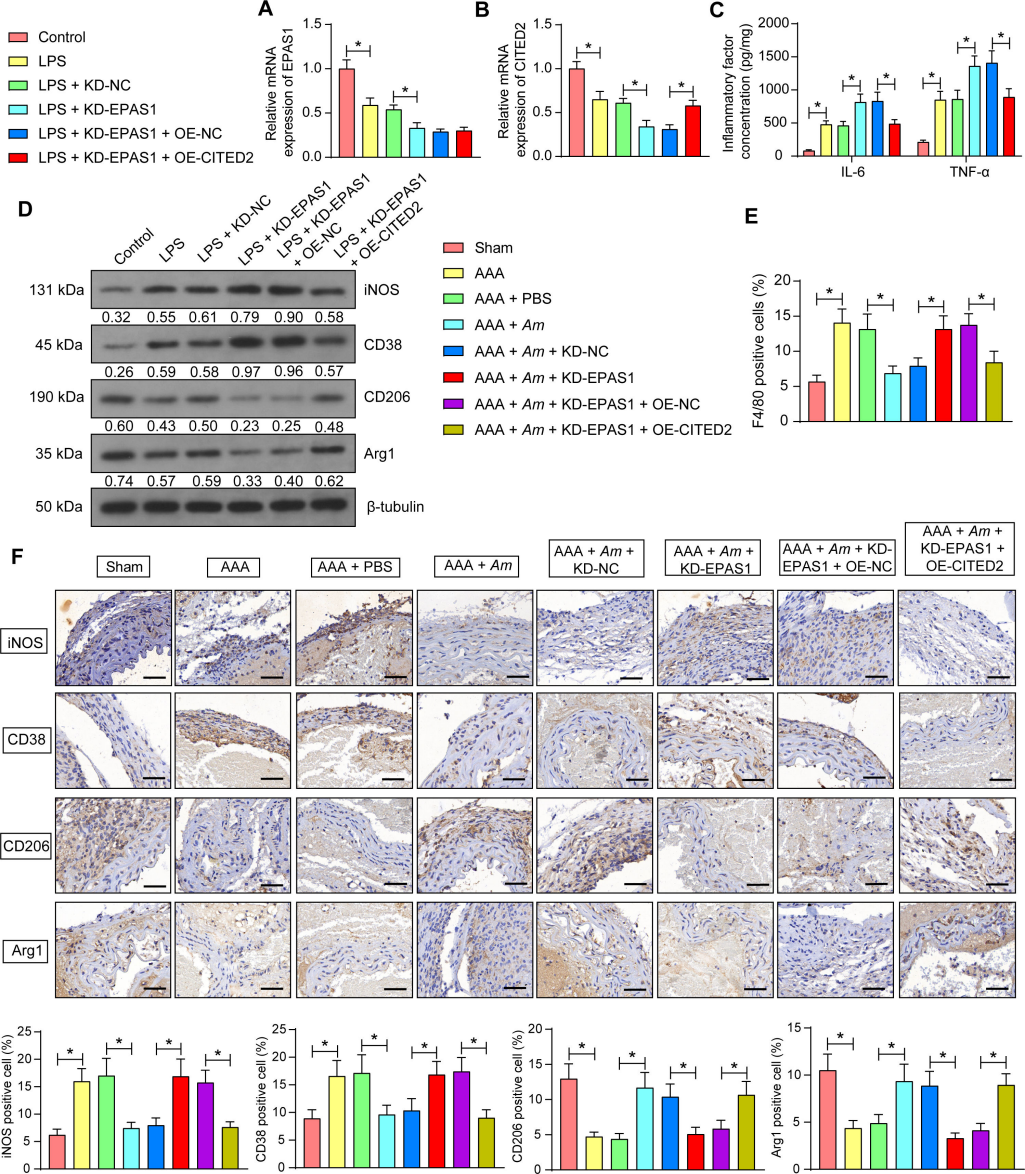
EPAS1 inhibits inflammatory response through upregulation of CITED2. Mouse macrophage J774A.1 cells were transfected with knockdown EPAS1 plasmid and overexpression CITED2 plasmid, followed by LPS induction. (**A**) The mRNA expression of EPAS1 in J774A.1 cells assessed using RT-qPCR (*n* = 3 independent experiments). (**B**) The mRNA expression of CITED2 in J774A.1 cells assessed using RT-qPCR (*n* = 3 independent experiments). (**C**) IL-6 and TNF-α levels in J774A.1 cells measured using ELISA (*n* = 3 independent experiments). (**D**) The protein expression of iNOS, CD38, CD206, and Arg1 in J774A.1 cell was measured using Western blot. (**E**) The expression of F4/80 in the abdominal aorta of mice with different treatments was observed using immunofluorescent staining. (**F**) The protein expression of iNOS, CD38, CD206, and Arg1 in the abdominal aorta of mice with different treatments was observed using immunohistochemistry. Scale bar, 50 µm. *n* = 6/group; *P < 0.05. Data were presented as mean ± SD and analyzed using one-way or two-way ANOVA.

To investigate the effect of CITED2 on the activation and pro-inflammatory response of macrophages, we transfected mouse macrophage J774A.1 cells with knockdown EPAS1 plasmid and overexpression CITED2 plasmid, followed by LPS induction. Knockdown of EPAS1 and overexpression of CITED2 were effective, as revealed by RT-qPCR (Fig. 7A and B). The concentrations of pro-inflammatory IL-6 and TNF-α were detected by ELISA. LPS was effective in inducing an inflammatory response, whereas the knockdown of EPAS1 further enhanced macrophage inflammatory response. However, overexpression of CITED2 inhibited the release of inflammatory factors from J774A.1 cells (Fig. 7C). Macrophage polarization markers iNOS, CD38, CD206, and Arg1 were detected by Western blot. Knockdown of EPAS1 enhanced the expression of M1 polarization markers iNOS and CD38 and reduced the expression of M2 polarization markers, CD206 and Arg1, whereas overexpression of CITED2 converted the macrophage to an M2 type of polarization (Fig. 7D).

The abdominal aortic tissues obtained from mice of the above eight groups were subjected to immunofluorescent staining. Macrophage infiltration could be observed in the abdominal aorta as a result of Ang II induction, which was attenuated by *Am* treatment. Knockdown of EPAS1 reversed the effect of *Am* on alleviating macrophage infiltration, and macrophage infiltration was reduced after overexpression of CITED2 (Fig. 7E). Immunohistochemistry was also conducted to examine the expression of four macrophage polarization markers. An increase in iNOS- and CD38-positive cells and a decrease in CD206- and Arg1-staining-positive cells were observed after Ang II treatment in mice. *Am* treatment decreased iNOS- and CD38-positive cells and increased CD206- and Arg1-positive cells, which was reversed by the knockdown of EPAS1. Still, overexpression of CITED2 decreased iNOS- and CD38-positive cells and increased CD206- and Arg1-positive cells in the presence of KD-EPAS1 (Fig. 7F).

## DISCUSSION

Subcutaneous infusion of Ang II, the primary bioactive peptide of the renin–angiotensin system, induces the development of AAAs in mice (12). By using 16S rRNA sequencing, we explored the alteration to the gut microbiome in Ang II-infused experimental AAA mice. In the present study, Ang II modeling contributed to a remarkable gut dysbiosis characterized by an altered gut microbial profile. Thus, these findings prompted us to determine how gut microbiota dysbiosis contributes to AAA development.

In response to vascular injury or alterations in local environmental cues, VSMCs switch to a dedifferentiated phenotype, characterized by extracellular matrix synthesis along with decreased expression of contractile markers, such as SM22 and α-SMA, and the loss of VSMC in the medial layer of the aortic wall because of apoptosis is an early hallmark of AAA development (13, 14). We observed these alterations in VSMC in the aortic tissues that showed pathological changes in mice induced with Ang II, indicating that the animal modeling was successful. Significant abnormalities were also identified in the gut microbe composition of AAA patients, indicating that gut microbiota dysbiosis is an important cause of AAA (15). Altered gut microbiota profiles and function have been observed in mice with AAA, with increased *Bacteroides* and *Parabacteroides* and reduced *Prevotella* and *Desulfovibrionaceae* (16). Consistently, we identified the reduced abundance of *Am* in the fecal samples from AAA mice. Administration with *Am* has been recently reported to diminish systolic blood pressure, promote fetal growth, and recover the placental pathology in mice with preeclampsia (17). In the present study, the gavage of *Am* alleviated the decline in VSMC contractile markers and inflammatory response in the aortic tissues. Subsequently, we predicted and verified EPAS1 as a vital mediator in the role of *Am* played in slowing AAA development.

Propionate, a main microbial fermentation metabolite in the human gut with putative health effects that extend beyond the gut epithelium (18), has been proposed to alleviate AAA by modulating colonic regulatory T-cell expansion and recirculation (19). Our *in vitro* evidence showed that the reduced expression of EPAS1 caused by Ang II in VSMC was restored following propionate treatment, further highlighting the regulatory effects of *Am* on EPAS1. It has been found that human cytomegalovirus infection inhibited proliferation but promoted VSMC apoptosis by upregulating microRNA-US33-5p, and microRNA-US33-5p bound to the 3’-untranslated region of EPAS1 to suppress its expression (20), indicating the role of EPAS1 in acute aortic dissection. Here, we found that the knockdown of EPAS1 further strengthened the exacerbating effects of Ang II on VSMC *in vitro* and *in vivo*, leading to enhanced cell apoptosis and inflammatory response and downregulated contractile marker expression.

Subsequently, CITED2 was found as a target of EPAS1 in AAA. The expression of CITED2 was reduced in VSMC and aortic tissues of mice induced by Ang II and further downregulated by EPAS1 loss. CITED2 has been identified as a negative regulator of macrophage pro-inflammatory activation that protects the host from inflammatory insults (21). Nevertheless, its role in VSMC, particularly under the context of AAA, has not been described previously. Our rescue experiments showed that the overexpression of CITED2 repressed the VSMC apoptosis, and inflammation in the aortic tissues of AAA mice in the presence of EPAS1 knockdown besides its direct role in narrowing the diameter of the abdominal aorta. Considering that AAA induced by Ang II presents the accumulation of macrophages, degradation of elastin, and thrombi in the media of the suprarenal aorta (22), we also examined the role of EPAS1 and CITED2 in macrophages in mice. Macrophages are major arsenals of the immune system against invaders (23). Beacuse plasticity and heterogeneity are hallmarks of macrophages, they are promising targets for therapies to reprogram toward specific phenotypes that could resolve disease and favor clinical prognosis (24). Several macrophage phenotypes have been described in inflamed tissues, with a particular focus on pro­inflammatory M1 macrophages and anti-inflammatory M2 macrophages (25). The *in vitro* and *in vivo* findings from LPS-induced J774A.1 cells and aortic tissues from mice yield consistent results.

**Fig 8:**
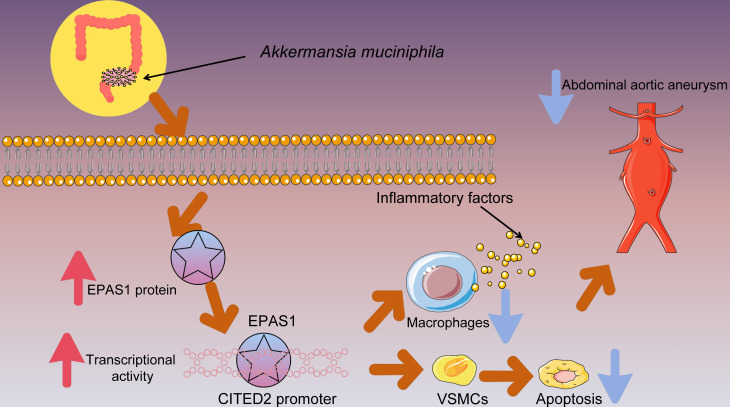
Schematic illustration. *Am* treatment promotes EPAS1 activation of CITED2 transcription, thereby inhibiting the pro-inflammatory response of macrophages and VSMC apoptosis to alleviate the development of AAA.

In conclusion, our research supported preliminary evidence that the regulatory role of the EPAS1/CITED2 axis in the pro-inflammatory response of macrophages and VSMC apoptosis in AAA is mediated by *Am* from the gut microbiota (Fig. 8).

## MATERIALS AND METHODS

### Mice

Forty-eight 8-week-old ApoE^-/-^ male mice were purchased from Cyagen (Suzhou, Jiangsu, China) and raised for 8 weeks in a temperature- and humidity-controlled room on a 12-/12-h light and dark cycle. The mice were fed on a high-fat diet (0.25% cholesterol and 15% cocoa butter). The mice were randomly divided into eight groups: sham, AAA, AAA + PBS, AAA + *Am*, AAA + *Am* + knockdown (KD)-negative control (NC), AAA + *Am* + KD-EPAS1, AAA + *Am* + KD-EPAS1 +overexpression (OE)-NC, and AAA + *Am* + KD-EPAS1 +OE-CITED2 groups (*n* = 6). For mice requiring lentivirus injection, they were subjected to tail vein injection of 100 µL of lentivirus-encapsulated knockdown of EPAS1 plasmid and overexpression of CITED2 plasmid (1 × 10^9^ PFU/mL). After 4 weeks, AAA mice were subcutaneously injected with phosphate-buffered saline (PBS) or Ang II (A9525, Sigma-Aldrich Chemical Company, St Louis, MO, USA) for 28 days (1.44 mg/kg/day).

*Am* (American Type Culture Collection, Manassas, VA, USA) was cultured anaerobically in brain heart infusion containing 1‰ cysteine at 37°C for 48 h and centrifuged at 6,000 rpm. The sample was resuspended in sterile PBS containing 25% glycerol, and the bacterial suspension was stored at −80°C. The mice were given 180 µL PBS or 2 × 10^8^ CFU *Am* by gavage daily after Ang II injection (6). Fecal samples were collected after 28 days and analyzed for differences in the abundance of gut microbiota in the feces of AAA mice vs sham-operated mice.

Immediately after *in vivo* measurement of aortic diameter by a Doppler ultrasound imaging system (DC-30, Mindary, Shenzhen, Guangdong, China), the mice were euthanized by intraperitoneal injection of sodium pentobarbital (150 mg/kg). The aorta was perfused with saline, and the peripheral connective tissues were removed. The aortic diameter was measured *ex vivo*, and maximum diameters 50% larger than those of the adjacent portion were defined as successful modeling (26). The abdominal aortic tissues were embedded in paraffin and used for subsequent experiments.

### 16s rRNA gene sequencing

Briefly, 200 mg of fecal samples was collected from each mouse. The DNA of the fecal samples was extracted and checked for purity and concentration, amplified by PCR according to the primers in the V3–V4 region of the 16s rRNA gene, and then sequenced by the Illumina MiSeq/HiSeq2500 High-Throughput Sequencing Platform.

### Culture and treatment of primary VSMC and J774A.1 cells

Mouse primary VSMC and mouse macrophage J774A.1 cells were purchased from Procell (Wuhan, Hubei, China), and the cells were maintained in DMEM containing 10% FBS, 100 U/mL penicillin, and 100 µg/mL streptomycin at 37°C with 5% CO_2_.

Overexpression of CITED2 plasmid and knockdown of EPAS1 plasmid were transfected into VSMC with Lipofectamine 2000 transfection reagent (11668019, Thermo Fisher Scientific Inc., Waltham, MA, USA) and cultured at 37°C, 5% CO_2_ for 48 h. After the medium renewal with fresh medium, the intervention effect of target genes was analyzed by RT-qPCR. VSMCs were induced with Ang II (10 µM) or PBS for 48 h. After Ang II induction, the cells were treated with PBS or 10 µg/mL propionate (P1880, Sigma-Aldrich) for 5 d (27).

Overexpression of CITED2 plasmid and knockdown of EPAS1 plasmid were transfected into macrophages with Lipofectamine 2000 transfection reagent (11668019, Thermo Fisher) and cultured at 37°C, 5% CO_2_ for 48 h. After the medium renewal with fresh medium, the intervention effect of target genes was analyzed using Western blot analysis. The macrophages were stimulated with 100 ng/mL of LPS (HY-D1056, MedChemExpress, Monmouth Junction, NJ, USA) for 2 h to induce macrophage inflammatory response (11).

### Hematoxylin–eosin (HE) staining

The abdominal aortic tissues of mice were stained with a HE staining kit (G1120, Beijing Solarbio Life Sciences Co., Ltd., Beijing, China). Paraffin-embedded sections were deparaffinized in xylene, rehydrated in ethanol (100%, 95%, 85%, 75%), stained with hematoxylin stain for 5 min, and washed with distilled water to remove the floating color. After that, the sections were stained with eosin stain for 2 min, dehydrated, cleared, sealed, and placed under the microscope for observation.

### Masson's staining

Mouse abdominal aortic tissues were stained according to the instructions of the Masson Staining Kit (G1340, Solarbio). Paraffin-embedded sections were deparaffinized, stained with Weigert’s iron hematoxylin staining solution for 5 min, stained with Ponceau 2R for 5 min, stained in aniline blue staining solution for 2 min, dehydrated in ethanol, cleared with xylene, and sealed with neutral gum for observation under the microscope.

### ELISA

Inflammatory factor levels in cell supernatants from VSMC or J774A.1 cells and abdominal aortic tissue homogenates were detected according to the instructions of the mouse IL-6 ELISA Kit (ab222503, Abcam Inc., Cambridge, UK), mouse TNF-α ELISA Kit (ab229393, Abcam), and IL-1β ELISA Kit (MLB00C, R&D Systems, Minneapolis, MN, USA).

### Western blot analysis

Mouse abdominal aortic tissues and VSMC were lysed with RIPA lysis buffer, and proteins were quantified by bicinchoninic acid protein assay kit (C503021, Shanghai Sangon Biological Engineering Technology & Services Co., Ltd., Shanghai, China). Proteins were separated by 10% SDS-PAGE and transferred to a PVDF membrane, which was sealed with 5% skimmed milk overnight at 4°C. These blots were further incubated with primary antibodies to SM22 (1:500, GTX636672, GeneTex, Inc., Alton Pkwy Irvine, CA, USA), α-SMA (1:1000, 19245, Cell Signaling Technologies, Beverly, MA, USA), Bcl-2 (1:2000, ab182858, Abcam), Cleaved-caspase-3 (1:1000, 9661S, Cell Signaling Technologies), iNOS (1:1000, ab283655, Abcam), CD38 (1:1000, 68336S, Cell Signaling Technologies), CD206 (1:1000, ab64693, Abcam), Arg1 (1:1000, GTX109242, GeneTex), β-tubulin (1:1000, ab179511, Abcam). After overnight incubation at 4℃, these blots were incubated for 1 h at room temperature in horseradish peroxidase-conjugated goat anti-rabbit secondary antibody (1:2000, ab205718, Abcam). Blots were visualized using enhanced chemiluminescence. The densitometry analyses were performed by utilizing ImageJ software.

### Apoptosis assay

VSMC apoptosis was detected by Annexin V Apoptosis Detection Kit (E606336, Sangon). VSMCs were resuspended in 195 µL 1 × binding buffer to reach a cell density of 2 × 10^5^ cells/mL, followed by the incubation with 5 µL Annexin V-FITC for 10 min at room temperature in the dark. The cells were rinsed with 200 µL 1 × binding buffer and centrifuged at 1,000 rpm for 2 min. After the removal of the supernatant, the cells were resuspended in 190 µL 1 × binding buffer, reacted with 10 µL propidium iodide, and loaded in flow cytometry within 4 h.

### Viability assay

VSMC viability was detected according to the instructions of cell counting kit-8 (CCK-8, E606335, Sangon). In brief, 2,000 cells (100 µL) were added to each well of a 96-well plate and incubated in an incubator at 37°C for 24 h. After a 1-h incubation with 10 µL CCK-8 solution with 5% CO_2_ at 37°C, the optical density (OD) value was measured at 450 nm.

### RNA extraction and RT-qPCR analysis

Trizol reagent (15596026CN, Thermo Fisher) was used to extract total RNA from VSMC and mouse aortic tissues. cDNA was synthesized using TaqMan Reverse Transcription Reagent (N8080234, Thermo Fisher), and qPCR was performed with ABsolute qPCR SYBR Green mixture (AB1285B, Thermo Fisher). The expression of target genes was determined by comparative ΔΔCt method, and GAPDH was used as an internal control gene. The primers used were as follows: EPAS1 (F 5′-GGACAGCAAGACTTTCCTGAGC-3′; R 5′-GGTAGAACTCATAGGCAGAGCG-3′), CITED2 (F 5′-TGCCGCCCAATGTCATAGACAC-3′; R 5′-AGAGTTCGGGCAGCTCCTTGAT-3′), STAT1 (F 5′-GCCTCTCATTGTCACCGAAGAAC-3′; R 5′-TGGCTGACGTTGGAGATCACCA-3′), STAT4 (F 5′-TCAGTGAGAGCCATCTTGGAGG-3′; R 5′-TGTAGTCTCGCAGGATGTCAGC-3′), EP300 (F 5′-GTGATGACCCTTCCCAACCTCA-3′; R 5′-CTCGTGGTGAAGGACACAGATC-3′), RUNX2 (F 5′-CCTGAACTCTGCACCAAGTCCT-3′; R 5′-TCATCTGGCTCAGATAGGAGGG-3′), GAPDH (F 5′-CATCACTGCCACCCAGAAGACTG-3′; R 5′-ATGCCAGTGAGCTTCCCGTTCAG-3′).

### Chromatin immunoprecipitation (ChIP)

Immunoprecipitation experiments were performed according to the instructions of the BeyoChIP ChIP Assay Kit (P2080S, Beyotime Biotechnology Co., Ltd., Shanghai, China). The cells were fixed with 1% formaldehyde and lysed with SDS lysis buffer containing protease inhibitors sufficiently. Sonication was performed to shear the genomic DNA, and 8 µL of 5 M NaCl was added to 0.2 mL of sonicated samples for 4-h incubation at 65°C to remove cross-links between proteins and genomic DNA. The samples were centrifuged at 4°C, 13,000 rpm for 5 min, and the supernatant (about 0.2 mL) was transferred into a 2-mL centrifuge tube and placed in an ice bath. The sonicated sample was diluted with 1.8 mL of ChIP dilution buffer containing protease inhibitor to a final volume of 2 mL. After that, 20 µL (1%) of the sample was collected as input for subsequent assays, and the remaining nearly 2 mL of sample was incubated with 50 µL of Protein A/G Magnetic Beads/Salmon Sperm DNA for 30 min at 4°C. ChIP was performed using antibodies to EPAS1 (1:50, 57921S, Cell Signaling Technologies) or isotype control IgG (2729S, Cell Signaling Technologies) at 4°C overnight. The complexes were precipitated with 80 µL of Protein A/G Magnetic Beads/Salmon Sperm DNA at 4°C for 60 min, which were placed on a magnetic rack for separation. Fluorescence qPCR was used to detect enrichment of the CITED2 promoter (chr10: 17722974–17723269) using the forward primer: 5′-GAGCCAGAAAGCCACTTCG-3′, reverse primer: 5′-CTGCCAACAATGAGCTGTGT-3′.

### Dual-luciferase reporter gene assay

A dual-luciferase reporter gene assay system was used to determine direct EPAS1 and CITED2 interactions. The promoter sequence of CITED2 was obtained from the UCSC Genome Browsers database (https://genome.ucsc.edu/), and potential binding sites for EPAS1 on the CITED2 promoter fragment were predicted by JASPAR. The WT CITED2 promoter sequence and the MT CITED2 promoter sequence (mutated at the EPAS1 binding site) were inserted into the pGL3-basic luciferase plasmid (Promega Corporation, Madison, WI, USA) using restriction endonuclease. The Lipofectamine 2000 transfection reagent was used to transfect the pGL3-basic luciferase plasmid with the knockdown EPAS1 plasmid into VSMC, and luciferase activity was detected at 48 h post-transfection.

### TUNEL assay

Mouse aortic tissues were stained according to the instructions of the TUNEL Kit (C1091, Beyotime). Paraffin-embedded sections were deparaffinized and treated with 20 µg/mL DNase-free proteinase K for 20 min at 37°C. PBS was used to wash away proteinase K, and the sections were incubated with 3% hydrogen peroxide for 20 min at room temperature to remove endogenous peroxidase and with 50 µL of biotin-labeling solution at 37°C for 60 min in the dark. After termination of labeling reaction termination solution for 10 min, the sections were incubated with 50 µL streptavidin-HRP working solution for 30 min and developed with diaminobenzidine (DAB) color development solution for 10 min (all at room temperature). The nuclei were stained with a hematoxylin staining solution. After dehydration in ethanol and clearing in xylene, the sections were subsequently sealed for observation.

### Immunofluorescence staining

Staining was performed using an immunofluorescence staining kit (P0176, Beyotime). Paraffin-embedded sections were deparaffinized, fixed in 4% paraformaldehyde for 10 min, sealed with immunostaining blocking solution ([P0102, Beyotime] for 60 min, and incubated with the primary antibody F4/80 (1:50, ab300421, Abcam) overnight at 4°C and with the fluorescence-labeled secondary antibody at room temperature in the dark for 60 min. The anti-fluorescence quenching sealing solution supplied in the kit was added dropwise to the sections that were observed under the fluorescence microscope.

### Immunohistochemical staining

Paraffin-embedded sections of aortic tissues were deparaffinized. The endogenous peroxidase was inactivated by 3% hydrogen peroxide. The sections were incubated with 10% fetal bovine serum to block nonspecific binding sites and with the primary antibodies to iNOS (1:2000, ab283655, Abcam), CD38 (1:100, 68336S, Cell Signaling Technologies), CD206 (1:10000, ab64693, Abcam), and Arg1 (1:1000, GTX109242, GeneTex) overnight at 4°C, followed by the secondary antibody incubation with biotin-labeled goat anti-rabbit IgG (1:50, SHB134, Solarbio) at 37°C for 30 min. Finally, the sections were visualized with DAB substrate, counter-stained, dehydrated, sealed, and microscopically observed.

### Quantitative and statistical analyses

Unless specifically indicated, all data were presented as the mean ± SD. For normally distributed data, an unpaired *t*-test was used to compare the differences between two groups, and one- or two-way ANOVA followed by Tukey’s *post hoc* analysis was used to compare three or more groups. A *P*-value <0.05 was considered significant. All results are representative of at least three independent experiments.

## Data Availability

Most of the data supporting our conclusions are included in the article; additional data may be found at https://figshare.com/s/32a416979039b571ae13. Further inquiries can be directed to the corresponding author.
